# A tomato a day keeps the beetle away – the impact of *Solanaceae* glycoalkaloids on energy management in the mealworm *Tenebrio molitor*

**DOI:** 10.1007/s11356-024-35099-4

**Published:** 2024-09-25

**Authors:** Magdalena Joanna Winkiel, Szymon Chowański, Karolina Walkowiak-Nowicka, Marek Gołębiowski, Małgorzata Słocińska

**Affiliations:** 1 Department of Animal Physiology and Developmental Biology, Institute of Experimental Biology, Faculty of Biology, Adam Mickiewicz University in Poznań, Uniwersytetu Poznańskiego 6, 61-614 Poznań, Poland; 2 Laboratory of Analysis of Natural Compounds, Department of Environmental Analytics, Faculty of Chemistry, University of Gdańsk, Wita Stwosza 63, 80-308 Gdańsk, Poland

**Keywords:** Citrate synthase, Phosphofructokinase, Metabolic pathway, Nutrients, Plant secondary metabolite, Glycoalkaloid

## Abstract

Solanine (SOL), chaconine (CHA), and tomatine (TOM) are plant secondary metabolites produced mainly by the species of *Solanaceae* family, such as tomato *Solanum lycopersicum* L. These glycoalkaloids (GAs) have a wide range of biological activity, also in insects. However, their mechanisms of action are not precisely understood. The purpose of the study was to investigate how pure GAs and tomato leaf extract (EXT) affect glycolysis, Krebs cycle and β-oxidation of fatty acid pathways in *Tenebrio molitor* L. beetle. For this purpose, the larvae were injected with SOL, CHA, TOM, and EXT at two concentrations (10^–8^ and 10^–5^ M). For experiments, fat body, gut, and heamolymph samples were collected 2 and 24 h after injection. Then, the changes in the expression level of phosphofructokinase, citrate synthase, and β-hydroxyacyl-CoA dehydrogenase were measured using the RT-qPCR technique. The catalytic activity of these enzymes and the carbohydrate level in insects after GA treatment were determined by spectrophotometric method. Furthermore, the analysis of the amount of amino acids in tissues was performed with a GC–MS technique. The results obtained show that the GAs changed the activity and expression of the genes encoding key enzymes of crucial metabolic pathways. The effect depends on the type of GA compound, the tissue tested, and the incubation time after treatment. Furthermore, TOM and EXT affected trehalose concentration in the insect hemolymph and led to accumulation of amino acids in the fat body. The observed changes may indicate a protein degradation and/or enhanced catabolism reactions for the production of ATP used in detoxification processes. These results suggest that GAs alter energy metabolism in the mealworm *T. molitor*. The study contributes to our understanding of the mechanisms of action of secondary metabolites of plants in insects. This knowledge may allow the design of new natural biopesticides against insect pests because proper energy metabolism is necessary for the survival of the organism.

**Supplementary Information:**

The online version contains supplementary material available at 10.1007/s11356-024-35099-4.

## Introduction

Glycoalkaloids (GAs) are plant secondary metabolites produced primarily by many *Solanaceae* plants, such as tomato *Solanum lycopersicum* L., potato *Solanum tuberosum* L., and eggplant *Solanum melongena* L. These compounds are composed of a steroidal carbon skeleton connected to 1–4 carbohydrates. For example, solanine (SOL) and chaconine (CHA) contain solanidine as an aglycon part, while tomatine (TOM) is built from a tomatidine skeleton. Carbohydrate chains in SOL, CHA and TOM are called solatriose, chacotriose, and lycotetraose, respectively (Zhao et al. 2021). GAs play a defensive role against various pathogens and herbivore species. These plant secondary metabolites exhibit a wide range of biological activities, such as anti-inflammatory, cytotoxic, and antimicrobial activity (Zhao et al. 2021). GAs disrupt cell membranes through binding to cholesterol molecules and inhibit acetylcholinesterase and butyrylcholinesterase enzymes. Moreover, these compounds impact the process of cell division, as well as the ion transport (Ca^2+^, Na^+^) across cell membranes (Friedman 2006; Milner et al. 2011). GAs may inhibit the growth of cancer cells, for example, by inhibiting angiogenesis, as well as apoptosis induction, because of their antiproliferative and pro-apoptotic activity. They affect many signalling pathways in tumour cells acting through different molecular mechanisms (Winkiel et al. 2022). Recent evidence suggests that SOL regulates glycolytic pathway in vitro in non-small cell lung cancer, decreasing the expression level of the genes encoding glycolysis-related proteins, such as glucose-6-phosphate isomerase, aldolase A and lactate dehydrogenase A (Zou et al. 2022), and in human renal cancer, reducing the expression of HIF-1α protein (Wang et al. 2021).

Insects store energy reserves in the form of glycogen and triglycerides (TAGs) in adipocytes, the fat body cells. Moreover, this tissue synthesizes most of the metabolites. Glycogen is a polymeric form of glucose, which is used as a glycolytic substrate and, for example, for chitin production. This polysaccharide is synthesized from dietary carbohydrates and amino acids. Glycogen is utilized mostly in the form of trehalose which is the main circulating sugar in the haemolymph. It is secreted into that tissue by adipocytes with cellular membrane transporters (Arrese and Soulages 2010). Glucose may be used for the synthesis of trehalose, glycogen and lipids. Fatty acids, which serve for ATP production during β-oxidation, are stored in the fat body in the form of TAGs, which are constituted of glycerol and three fatty acid molecules. Trehalose or proline, by conversion into diglyceride, can be used in some insects as a key energetic substrates in flight muscle (Arrese and Soulages 2010). The proline amino acid is produced in the fat body from acetyl-CoA and alanine and is released to the haemolymph. This amino acid synthesis is often connected to the fatty acid β-oxidation because inhibition of β-oxidation blocks the release of trehalose induced by adipokinetic hormone (Bursell 1981; Arrese and Soulages 2010). In general, insects were found to contain higher amino acid amounts compared to the other animal species. Amino acids are utilized for proteins production, therefore, they fulfill structural and developmental functions (Chen 1966). The following amino acids: arginine, histidine, lysine, tryptophan, phenylalanine, methionine, threonine, leucine, isoleucine, and valine considered essential for mammals are also necessary for the growth of *Tenebrio molitor* L. larvae (Chen 1966; Davis 1975).

The main substrate for glycolysis is glucose, which is converted into pyruvate during the glycolysis that occurs in the cytosol. In this pathway, two ATP moieties are generated. One of the three key regulatory glycolysis reactions is the process catalyzed by phosphofructokinase-1 (PFK). This enzyme is necessary for irreversible phosphorylation of fructose 6-phosphate to fructose 1,6-bisphosphate. The reaction is regulated by a feedforward activation mechanism, as well as by citrate, the intermediary metabolite of the Krebs (tricarboxylic acid –TCA) cycle. Some glycolytic intermediates can enter other biosynthetic pathways. For example, the product of the reaction catalyzed by PFK can be converted to dihydroacetone phosphate, which, in turn, in the next step is transformed into glycerol 3-phosphate, a substrate for TAGs production (Chandel 2021a). In the presence of oxygen, pyruvate is usually oxidized to acetyl-CoA during oxidative decarboxylation and then converted to two CO_2_ moieties in the Krebs cycle which occurs in mitochondria. The reaction of citrate synthesis is catalyzed by citrate synthase (CS) which is a marker enzyme of the TCA cycle, at the gateway into the cycle from pyruvate via acetyl-CoA. Besides the pyruvate generated in the glycolysis process, fatty acids can be used as the substrate for the TCA cycle. These compounds are the main source of ATP during the low glucose level in the cell. One of the steps of β-oxidation of fatty acids is the conversion of L-β-hydroxyacyl-CoA to β-ketoacyl-CoA, catalyzed by β-hydroxyacyl-CoA dehydrogenase (HADH) (Chandel 2021b).

As glycolysis, Krebs cycle, and β-oxidation of fatty acids are important processes of ATP production in cells, the question is, if GAs can affect these reactions. The first study on the effect of GAs on blood sugar levels was reported already in 1967 year (Satoh 1967). At that time, it was predicted that SOL may act as a hyperglycemic agent in rats. However, later this issue did not attract interest among scientists, who focused on other effects caused by GAs. In insects, GAs were found to alter the functioning of numerous processes from feeding through reproduction to behaviour (Chowański et al. 2016). Only recently have some relationships between GAs and lipid metabolism been established. We have previously reported that the application of GAs and tomato leaf extract into *T. molitor* larvae affects the content and composition of lipid compounds in the insects' haemolymph and fat body. Furthermore, HADH activity decreased after GA application, especially in the fat body, which may affect ATP production (Winkiel et al. 2023). After treatment with solamargine, solasonine and *Solanum nigrum* L. extract, a loss of homogeneity of lipid droplets and their regularity of shape were observed in the *T. molitor* beetle and *Galleria mellonella* L. moth (Spochacz et al. 2018, 2021). These compounds also affected the ultrastructure of midgut cells, as well as carbohydrate, lipid, and amino acid content in the fat body and haemolymph of insects (Spochacz et al. 2018, 2021). However, it is still unknown which mechanisms underlie these metabolic fluctuations in insects. We do not know if the observed changes are a result of the impact of GAs on the level of genes encoding crucial enzymes of metabolic pathways or their influence on the protein level.

ATP production is necessary for the survival of cells and the entire organism. Thus, the study aimed to verify if SOL, CHA, TOM, and EXT may alter key steps of glycolysis, Krebs cycle and β-oxidation of fatty acids at the gene and protein levels, and the content of energy substrates in insect tissues. Despite recent interest in *T. molitor* larva-based food products, it is a pest that cause significant losses in grain stores, and therefore its control is necessary in certain environments. It is especially important to explore the effects of GAs on energy-producing processes in insects, because umbalancing of energetic homeostasis can impact the condition of individual insects and, in consequence, reduce the population of harmful species (Manosathiyadevan et al. 2017). The obtained data extends the knowledge about GAs mechanisms of action that is necessary to consider these compounds as potential promising biopesticides.

## Materials and methods

### Insects

*Tenebrio molitor* beetles were cultured at the Department of Animal Physiology and Developmental Biology at the Faculty of Biology of Adam Mickiewicz University in Poznań, Poland at constant temperature (26 ± 0.5 °C), humidity (65 ± 5%) and photoperiod 8:16 h light to dark, as described previously (Winkiel et al. 2023). The food consisted of oat flakes and fresh carrots. In experiments, only feeding larvae from the 15th to 16th instar of approximately 120 to 140 mg of weight were used.

### Compounds and treatment procedure

In experiments, synthetic GAs: SOL (≥ 95.0%), CHA (≥ 95.0%), and TOM (≥ 95.0%) (Merck Sigma-Aldrich) in a form of saline solutions were used. The concentrations applied were as follows: 10^−8^ M (dosage range for SOL and CHA 0.12–0.14 pg/mg body mass, for TOM 0.15–0.17 pg/mg body mass) and 10^−5^ M (dosage range for SOL and CHA 0.12–0.14 ng/mg body mass, for TOM 0.15–0.17 ng/mg body mass) (Winkiel et al. 2023). The concentrations of GAs were selected based on the literature and our previous studies in which we observed that application of these compounds led to various metabolic and developmental changes (Spochacz et al. 2018, 2021; Winkiel et al. 2023). The GA extract from tomato leaves (EXT) is acetic acid aqueous solution. It was obtained from the research group of Prof. Sabino A. Bufo from Basilicata University in Potenza, Italy, and tested previously by our group (Ventrella et al. 2015, 2016; Marciniak et al. 2019; Winkiel et al. 2023). EXT contained TOM, dehydrotomatine and filotomatine (Ventrella et al. 2016). TOM concentration in the EXT corresponded to its content in 10^−8^ and 10^−5^ M solutions of this GA. Thus, it was possible to compare the effects of pure GA and its extract. The control was the physiological solution for *T. molitor* (Winkiel et al. 2023)*.* The tested compounds were administered to insects by injection using a microsyringe (Hamilton) in a volume of 2 μL, as described previously (Winkiel et al. 2023). The injection was made after 8 min of CO_2_ anaesthesia. The final concentration in the haemolymph was 10^−9^ and 10^−6^ M.

### Tissue isolation

The tissue isolation was performed 2 or 24 h after GA injection, according to the method described previously (Winkiel et al. 2023). Briefly, the trophic tissues (haemolymph, gut, and fat body) that play a key role in maintaining metabolic balance, as well as detoxification, were isolated using the microsurgical tools after 8 min of anaesthesia with CO_2_. To avoid degradation of the samples, the isolation was carried out on ice. The samples were stored at − 80 °C until the next steps.

### Concentration of carbohydrates

The analysis of glucose, trehalose, and glycogen levels was performed using samples prepared as previously described for triacylglyceride (TAG) determination (Winkiel et al. 2023). The samples were pooled from two individuals. The isolated samples of fat body tissue (about 46 mg) and haemolymph (16 μL) were next homogenized on ice in 300 μL and 150 μL of PBS-Tween 0.05%, respectively, using a pestle homogenizer. Then, after incubation at 70 °C for 10 min, the samples were centrifuged (10,000 RPM, 5 min, 4 °C), and the supernatant was transferred to new tubes. For each experimental variant, four independent replicates were performed. The samples were frozen in liquid nitrogen and stored at − 80 °C until the measurements were made.

The tested carbohydrates were determined spectrophotometrically in undiluted haemolymph samples. The fat body samples were used undiluted for glucose analyses, while diluted tenfold with PBS-Tween 0.05% for trehalose and glycogen level determination. For glucose level analyses, the Glucose Assay Kit (Merck Sigma-Aldrich; GAGO20) was used according to the manufacturer’s protocol. Each sample (15 μL) and 50 μL of the Assay Reagent were placed on a clear-bottom 96-well plate and incubated at 37 °C for 60 min. Then, 50 μL of sulfuric acid (Merck Sigma-Aldrich; 339,741) was added as a reaction inhibitor. After 10 min, the absorbance was measured at wavelength λ = 540 nm at RT with a Synergy H1 Hybrid MultiMode Microplate Reader (BioTek). The glucose level in each sample was calculated using the standard curve. However, it was not detected in haemolymph, and it was very low glucose concentration in fat body samples. Therefore, it was neglected, and the concentration of trehalose was determined after the addition of trehalase (Merck Sigma-Aldrich; T8778-1UN) to the Assay Reagent (1:1000); for glycogen analyses, aminoglucosidase (Merck Sigma-Aldrich; A1602) was added to the Assay Reagent (3:1000). The standards of trehalose (Merck Sigma-Aldrich; T9449) and glycogen (Merck Sigma-Aldrich; G8751) were used to prepare the standard curves. The carbohydrate level is expressed in µg per 1 mg of fresh tissue.

### Concentration of amino acids

The level of amino acids in the samples of haemolymph and fat body was evaluated using gas chromatography‒mass spectrometry (GC‒MS). In experiments, pooled samples were used with n ≥ 15 (haemolymph) or n ≥ 10 (fat body). After isolation, tissues were placed into glass bottles with chloroform and methanol 2:1 (v/v) and kept at 4 °C.

Amino acids were determined with the GC‒MS technique according to the method described previously (Szymczak-Cendlak et al. 2022; Winkiel et al. 2023). Briefly, amino acids were extracted in 30 mL of dichloromethane. The solvent was removed from the samples under a gentle stream of nitrogen. Components of extracts were silylated with 100 μL of a mixture of 99% bis(trimethylsilyl)acetamide and 1% chlorotrimethylsilane at 100 °C for 1 h on the day of analysis. The samples were analyzed using GC–MS on a GC/MS QP2010 SE (Shimadzu, Kyoto, Japan) equipped with a fused silica capillary column Zebron–5, 30 m × 0.25 mm i.d. and with a 0.25 µm thick film. Helium was used as the carrier gas. The ion source was maintained at 220 °C. The injector and transfer line temperatures were kept at 310 °C. Electron-impact ionization (electron energy 70 eV) was used. The column temperature was programmed at 4 °C × min − 1 from 80 (held for 10 min) to 310 °C, which was held for 10 min. The amino acid level is expressed in µg per 1 mg of fresh tissue. The analyses were performed in triplicate.

### Quantitative analysis of gene expression

The samples for gene expression measurements were pooled from 5 individuals. Tissues (fat body and gut) were placed into 300 μL of RNA Lysis Buffer (Zymo Research; R1060-1), homogenized for 3 min using a pestle homogenizer (Fisherbrand), and the total RNA isolation was conducted using Quick-RNA™ MiniPrep Kit (Zymo Research; R1055), according to the manufacturer's protocols. The residual DNA was then removed with a Turbo DNase kit (Thermo Scientific; AM1907), and the RNA concentration was measured spectrophotometrically (DeNovix DS-11 FX +). After that, the RNA samples were frozen in liquid nitrogen and stored at − 80 °C until the next steps.

The synthesis of cDNA was conducted using LunaScript® RT SuperMix Kit (Biolabs; E3010) and T100™ Thermal Cycler (BIO-RAD). The prepared cDNA samples were stored at − 20 °C. Quantitative real-time PCR (RT-qPCR) analyses were performed with a SYBR Green Master mix (Thermo-Fisher Scientific; 4,309,155) on a C1000™ Thermal Cycler with the CFX96™ Real-Time System (BIO-RAD). The primers were designed using Primer3plus software based on sequences available in public databases (NCBI; CS: XM_965031.5, HADH: XM_967949.5, PFK: XM_961686.4) and synthesized by the Institute of Biochemistry and Biophysics, Warsaw (Supp. Mat.). The suitability of the primers for the qPCR was tested by analyzing the melting curves. The PCR conditions for the amplified gene and the reference gene (ribosomal protein L13a (Rpl13a)), were determined and optimized before amplification. The stability of *Rpl13a* expression was validated prior to the experiment. The experiment was prepared in three biological replicates and three independent replicates for each experimental variant. Negative controls were prepared to check for possible contamination of the samples. Relative expression was calculated using the 2^ΔΔ^Ct method (Livak and Schmittgen 2001). To confirm the results, the amplicons were sequenced by the Molecular Biology Techniques Laboratory (Faculty of Biology, Adam Mickiewicz University in Poznań) and compared with the data available in a public database (NCBI).

### Enzyme activity

The activity of PFK and CS in the gut and fat body was measured in samples pooled from a minimum of 10 individuals, according to the method described previously (Winkiel et al. 2023). Briefly, the tissues were placed in 250 μL (gut) or 500 μL (fat body) of physiological saline, homogenized, and centrifuged. The protein concentration was measured using a Direct Detect spectrometer (Merck) (Szymczak-Cendlak et al. 2022). Then, the samples were frozen in liquid nitrogen and stored at − 80 °C until the next steps.

The PFK and CS catalytic activity was measured using commercially available kits (Merck Sigma-Aldrich; MAK093 and MAK193, respectively). The experiment was carried out according to the manufacturer's instructions. The gut samples for the experiment were diluted 16x (PFK) or 10x (CS). On the contrary, fat body samples were diluted to a total protein concentration of 3.0–4.2 µg/μL with 4 mM kojic acid in PBS buffer. Then, the samples were put on the plate (PFK) or diluted again with the kit buffer 50x (CS). Kojic acid was used as a polyphenol oxidase inhibitor to reduce the interference of the polyphenol oxidase reaction product with the product of the reaction catalysed by the tested enzymes. The experiments were based on the spectrophotometric technique using Spark Microplate Reader (Tecan, Switzerland). The absorbance was measured at wavelength λ = 450 nm (PFK) and λ = 412 nm (CS) at RT for 50 min (5 min intervals). Enzyme activity is expressed as mU per μg of total soluble protein in the sample. The assays were prepared in three independent replicates for each experimental variant.

### Statistical analysis

The results were analysed using Graphpad Prism 8.0.1. (Department of Animal Physiology and Developmental Biology AMU license). The normality of the distribution was determined using the Shapiro–Wilk test. Normally distributed data were analysed with ordinary one-way ANOVA or Brown–Forsythe and Welch ANOVA with Dunnett's multiple comparison tests. Data with a non-normal distribution were analysed using Kruskal–Wallis with Dunn's multiple comparison tests. In graphs, the data are presented as a mean ± SEM (Standard Error of the Mean).

## Results

### Level of carbohydrates

The concentration of glucose, trehalose, and glycogen was analysed in fresh tissue of fat body and haemolymph after GAs injections at two concentrations 10^–8^ and 10^–5^ M.

**Fig. 1:**
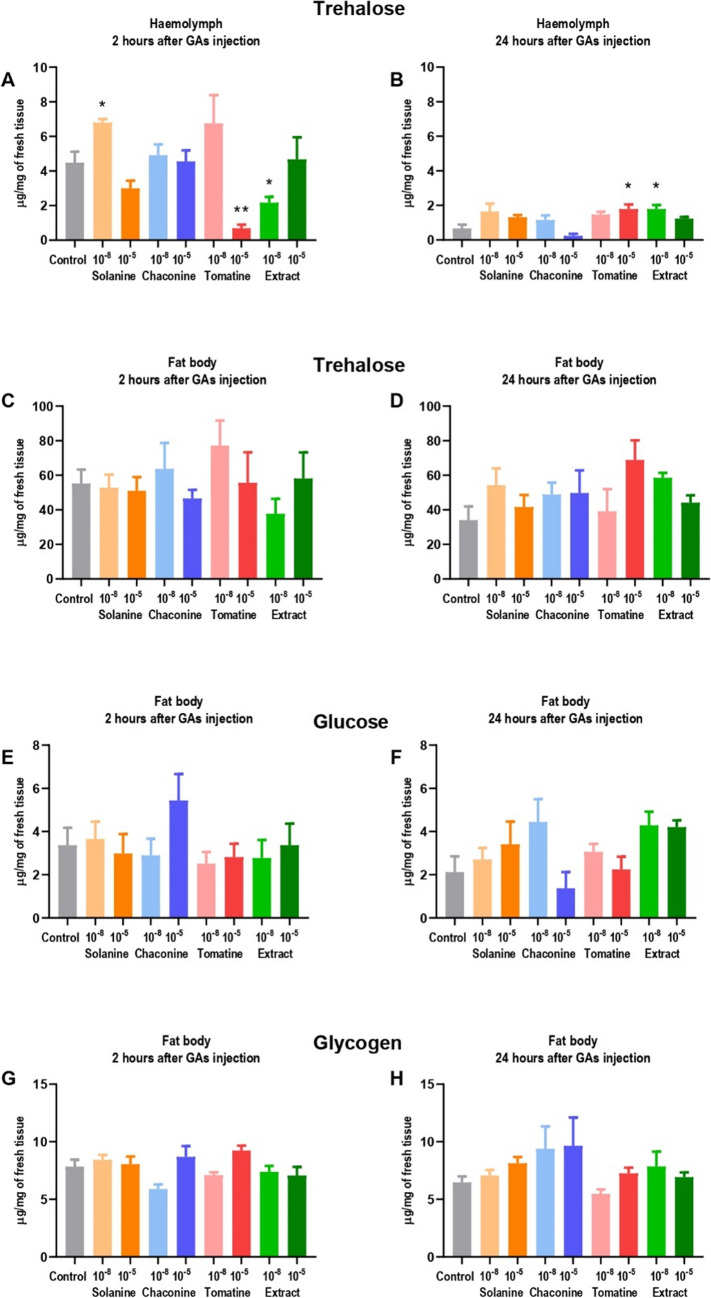
Concentration of trehalose (**A-D**), glucose (**E**, **F**) and glycogen (**G**, **H**) in the fat body and haemolymph of *Tenebrio molitor* larvae 2 and 24 h after injection with glycoalkaloids and physiological saline (control). Concentrations of the compounds 10^−8^ M and 10^−5^ M are presented above (10^−8^, 10^−5^). The results are expressed in µg per mg of fresh tissue. Four independent replicates using pooled samples were performed. Data are presented as mean ± SEM and compared with Brown–Forsythe and Welch ANOVA with Dunnett’s multiple comparison tests. Statistically significant differences between tested groups and the control are indicated with asterisks, ** *p* ≤ 0.01, * *p* ≤ 0.05

The trehalose concentration in the haemolymph 2 h after GAs treatment ranged from 0.7 ± 0.60 to 6.8 ± 0.58 µg per 1 mg of tissue (Fig. 1A), while in 24 h variant, there was between 0.2 ± 0.03 and 1.8 ± 0.66 µg of trehalose per 1 mg (Fig. 1B). SOL at lower concentration (10^–8^ M) significantly increased the disaccharide level in haemolymph after 2 h. On the contrary, a decrease in trehalose content was observed 2 h after TOM 10^–5^ M, as well as after the application of EXT 10^–8^. Interestingly, in these experimental variants, an increase in disaccharide concentration was observed 24 h after GA treatment. In the fat body, the trehalose concentration in the 2 h variant was between 37.7 ± 24.68 and 77.2 ± 41.01 µg per 1 mg (Fig. 1C), and 24 h after GAs application it ranged from 39.1 ± 36.66 to 68.9 ± 32.14 µg per 1 mg of fresh tissue (Fig. 1D). No significant changes in the content of this carbohydrate were reported neither 2, nor 24 h after GA treatment.

The glucose level in the fat body tissue did not change compared to the control, neither 2 (Fig. 1E) nor 24 h (Fig. 1F) after GA injection. The calculated glucose concentration in the samples was between 1.4 ± 2.12 and 5.4 ± 3.45 µg per 1 mg of fresh fat body tissue. There was a slight increase in glucose content 2 h after CHA 10^–5^ M treatment, however, the change was not significant. On the other hand, 24 h after the GAs application, in this experimental variant, the monosaccharide concentration tended to decrease. In haemolymph, the amount of glucose was below the detection limit.

The glycogen concentration in the fat body did not change as a result of GAs injection, compared to the control (Fig. 1G, H). It ranged between 5.9 ± 1.12 – 9.2 ± 1.18 µg/mg in the case of 2 h variant, and between 5.5 ± 1.10 and 9.7 ± 6.96 µg per 1 mg of fresh tissue 24 h after treatment. No amount of glycogen was detected in the haemolymph samples.

### Level of amino acids

**Fig. 2:**
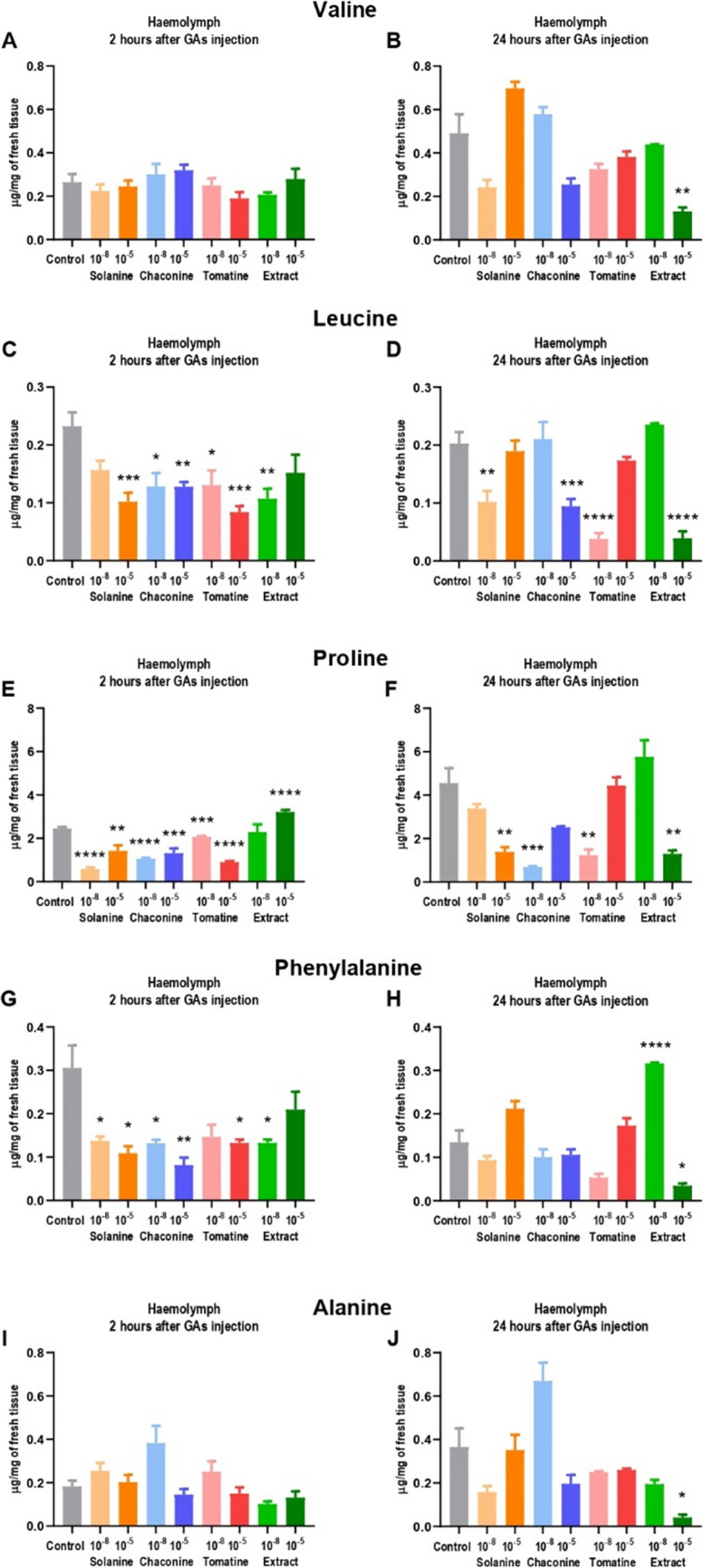
The concentration of amino acids in the haemolymph of *Tenebrio molitor* larvae 2 and 24 h after injection with glycoalkaloids and physiological saline (control). Concentrations of the compounds 10^−8^ M and 10^−5^ M are presented above (10^−8^, 10^−5^). The results are expressed in µg per mg of fresh haemolymph tissue and shown as the mean ± SEM. Pooled samples were used with* n* ≥ 10, and the analysis was performed in triplicate. Data are compared with Brown–Forsythe and Welch ANOVA with Dunnett’s multiple comparison tests. Statistically significant differences between tested groups and the control are indicated with asterisks, **** *p* ≤ 0.0001, *** *p* ≤ 0.001, ** *p* ≤ 0.01, * *p* ≤ 0.05

**Fig. 3:**
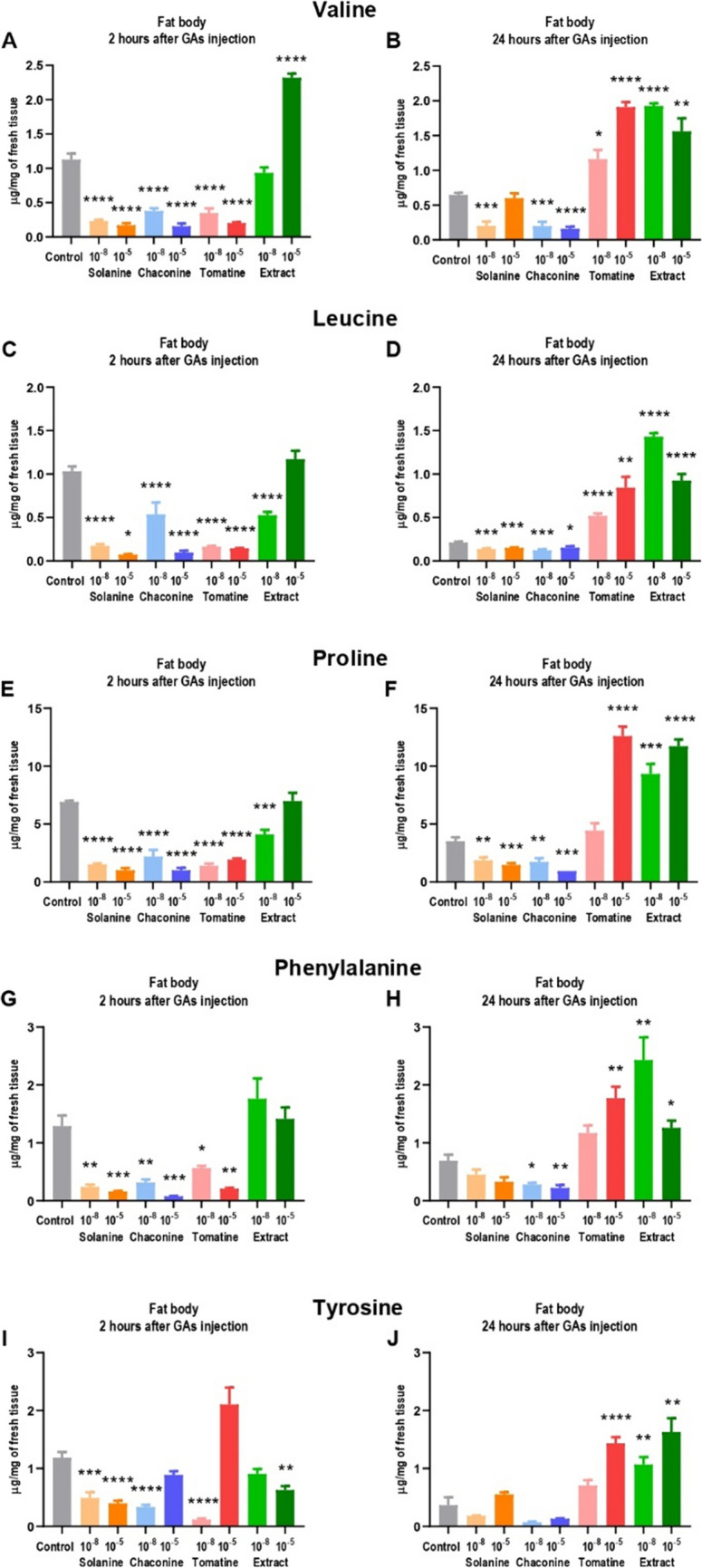
The concentration of amino acids in the fat body of *Tenebrio molitor* larvae 2 and 24 h after injection with glycoalkaloids and physiological saline (control). Concentrations of the compounds 10^−8^ M and 10^−5^ M are presented above (10^−8^, 10^−5^). The results are expressed in µg per mg of fresh fat body tissue and shown as the mean ± SEM. Pooled samples were used with* n* ≥ 10, and the analysis was performed in triplicate. Data are compared with Brown–Forsythe and Welch ANOVA with Dunnett’s multiple comparison tests. Statistically significant differences between tested groups and the control are indicated with asterisks, **** *p* ≤ 0.0001, *** *p* ≤ 0.001, ** *p* ≤ 0.01, * *p* ≤ 0.05

The following amino acids were detected in the tested sample of haemolymph and fat body: valine, leucine, proline, phenylalanine, alanine (only in haemolymph), and tyrosine (only in the fat body). The valine concentration in haemolymph 2 h after GAs injection did not change (Fig. 2A). It ranged between 0.2 ± 0.11 µg/mg and 0.3 ± 0.10 µg/mg of tissue. 24 h from the GAs application (Fig. 2B), the valine content was more differentiated (0.1 ± 0.07 – 0.7 ± 0.12 µg/mg). In this experimental variant, only a higher EXT concentration significantly decreased valine content in that tissue. In the fat body, all pure GAs decreased amino acid concentration during 2 h (Fig. 3A) with the greatest change after CHA 10^–5^ M treatment (more than sevenfold). On the contrary, EXT 10^–5^ M increased the valine concentration 2-times (2.3 ± 0.19 µg/mg) compared to the control (1.1 ± 0.28 µg/mg). 24 h after treatment with SOL 10^–8^ M, CHA 10^–8^ M, and CHA 10^–5^ M, the amino acid content in the fat body was still decreased compared to the control (Fig. 3B). However, TOM and EXT increased the valine concentration in the fat body. It indicates the possibility of amino acid transfer from haemolymph to that tissue.

Leucine concentration was decreased in the haemolymph in most of the 2 h experimental variants compared to the control (Fig. 2C). The greatest change was reported after TOM 10^–5^ M application (almost a threefold decrease). 24 h after GAs injections, the lower amino acid concentration was observed after 10^–8^ M SOL and TOM, as well as after 10^–5^ M CHA and EXT treatments (Fig. 2D). The application of all of the tested GAs after 2 h resulted in decreased leucine concentration in the fat body, except for EXT 10^–5^ M, which did not affect amino acid content compared to the control (Fig. 3C). Leucine concentration was decreased also 24 h after SOL and CHA application (Fig. 3D). However, similarly to valine, TOM as well as EXT after 24 h significantly increased amino acid content in the fat body compared to the control (even almost sevenfold).

All of the tested GAs, except for EXT 10^–5^ M, decreased proline concentration during 2 h in the haemolymph (Fig. 2E). The lowest amino acid content was reported after SOL 10^–8^ treatment (change from 2.5 ± 0.27 µg/mg in the control to 0.6 ± 0.30 µg/mg after GA injection). On the contrary, treatment with EXT 10^–5^ M after 2 h resulted in increased proline concentration in the haemolymph. 24 after injection, 10^–8^ M CHA and TOM, as well as 10^–5^ M SOL and EXT, maintained a decrease in proline concentration compared to the control (Fig. 2F). In the fat body, all pure GAs as well as EXT 10^–8^ M after 2 h caused a decrease in amino acid content (Fig. 3E). Similarly to the other amino acids, proline concentration in the fat body was lower 24 h after treatment with SOL and CHA (even almost 4 times), while higher compared to the control (also almost 4 times) after TOM and EXT injections (Fig. 3F).

The phenylalanine concentration in the haemolymph 2 h after the application of pure GAs and EXT 10^–8^ M was reduced compared to the control (Fig. 2G). The lowest amino acid value was noted after CHA 10^–5^ M injection (0.1 ± 0.07 µg/mg compared to the 0.3 ± 0.20 µg/mg in the control). Surprisingly, 24 h after application, EXT 10^–8^ M caused an increase in phenylalanine concentration in the haemolymph (more than 2-times), while the treatment with EXT 10^–5^ M resulted in a decrease in amino acid content (more than 4-times) compared to the control (Fig. 2H). Phenylalanine concentration in the fat body was decreased 2 h after pure GAs injections, while no change was observed after EXT application (Fig. 3G). 24 h after CHA injection, the amino acid content in the fat body remained decreased compared to the control, while it increased after TOM and EXT treatment (Fig. 3H). For example, the phenylalanine concentration was 0.7 ± 0.32 µg/mg in the control and 1.8 ± 0.64 µg/mg after TOM 10^–8^ M injection (2.6-fold change).

Alanine was detected only in the haemolymph. However, any of the tested GAs affected its concentration which ranged between 0.1 ± 0.05 µg/mg and 0.4 ± 0.31 µg/mg (Fig. 2I). Alanine concentration was significantly lower compared to the control only 24 h after the injection of EXT 10^–5^ M (Fig. 2J). In the other experimental variants, no changes were reported.

Tyrosine was reported only in the fat body. Most of the tested GAs decreased its concentration after 2 h (Fig. 3I). The biggest change was calculated after TOM 10^–8^ M treatment (tenfold decrease). 24 h after TOM 10^–5^ M and EXT injections, an increase in tyrosine concentration in the fat body was reported (Fig. 3J), similarly to the other amino acids.

### Quantitative analysis of gene expression

**Fig. 4:**
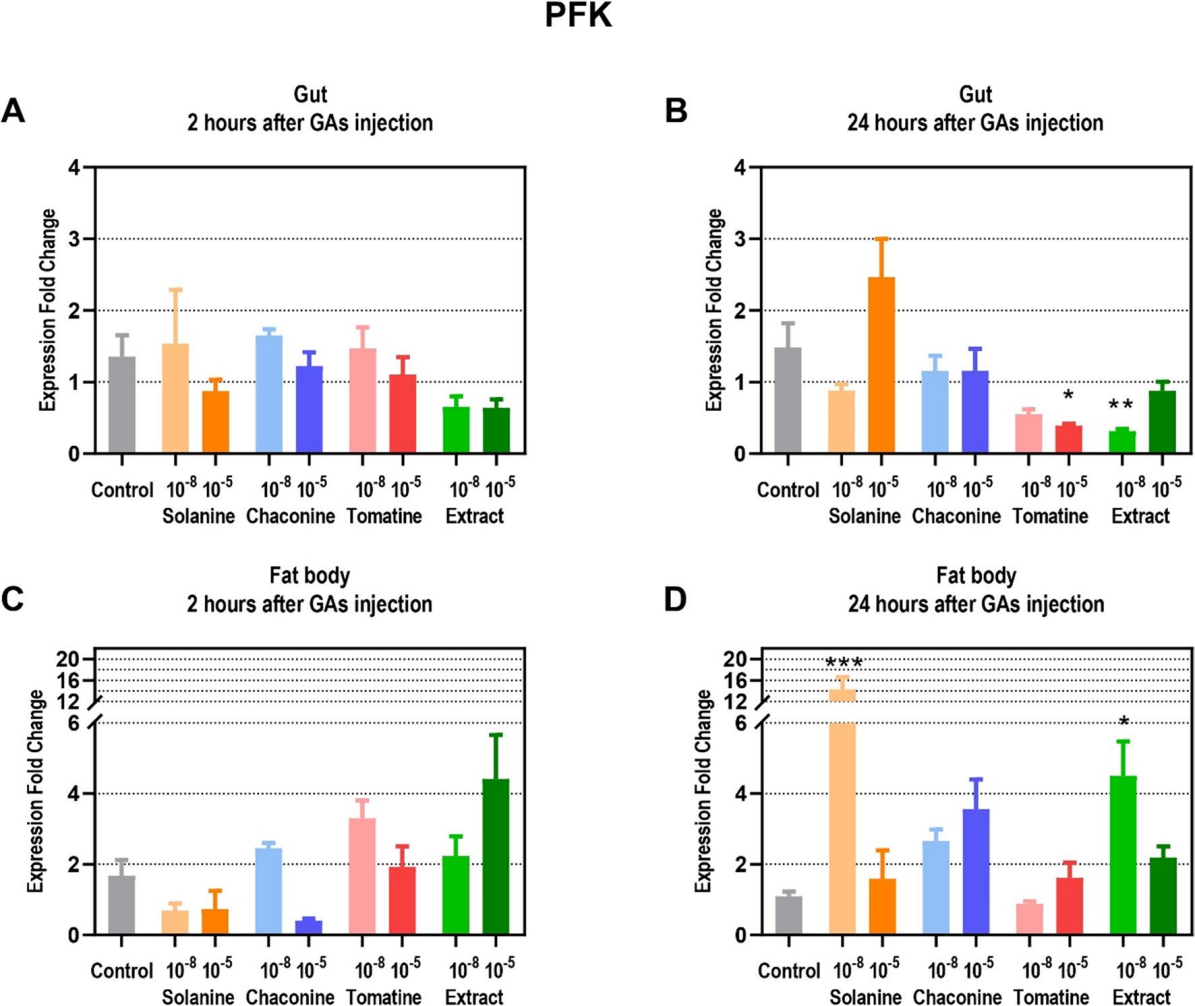
Expression fold change of phosphofructokinase-1 (PFK) in a gut (**A**, **B**) and fat body (**C**, **D**) of *Tenebrio molitor* larvae 2 and 24 h after application of 10^–8^ and 10^–5^ M solutions of glycoalkaloids and physiological saline (control) compared to the L ribosomal proteins (RPL) expression. Data are shown as mean ± SEM. The analysis was performed in triplicate. The pooled samples were used with *n* = 5. Data are compared with Kruskal–Wallis with Dunn's multiple comparison test. Statistically significant differences between tested groups and the control are indicated with asterisks, *** *p* ≤ 0.001, ** *p* ≤ 0.01, * *p* ≤ 0.05

The expression fold change of genes encoding PFK, CS, and HADH was calculated after GAs and physiological saline (control) injections in the gut and the fat body of insects. The expression of *PFK* did not change in the gut 2 h after the injection of all GAs tested (Fig. 4A). Only after 24 h after GAs application, TOM 10^–5^ M and EXT 10^–8^ M decreased the expression of the PFK genes in the gut almost 4– and almost fivefold, respectively, compared to the control (Fig. 4B). 2 h after GAs treatment, similar to the gut, also in the fat body there were no changes of *PFK* expression (Fig. 4C). However, 24 h after treatment, an increase in *PFK* expression was observed in this tissue after injection of SOL 10^–8^ M (13 times), as well as after EXT 10^–8^ M (over 4-times compared to the control) application (Fig. 4D).

**Fig. 5:**
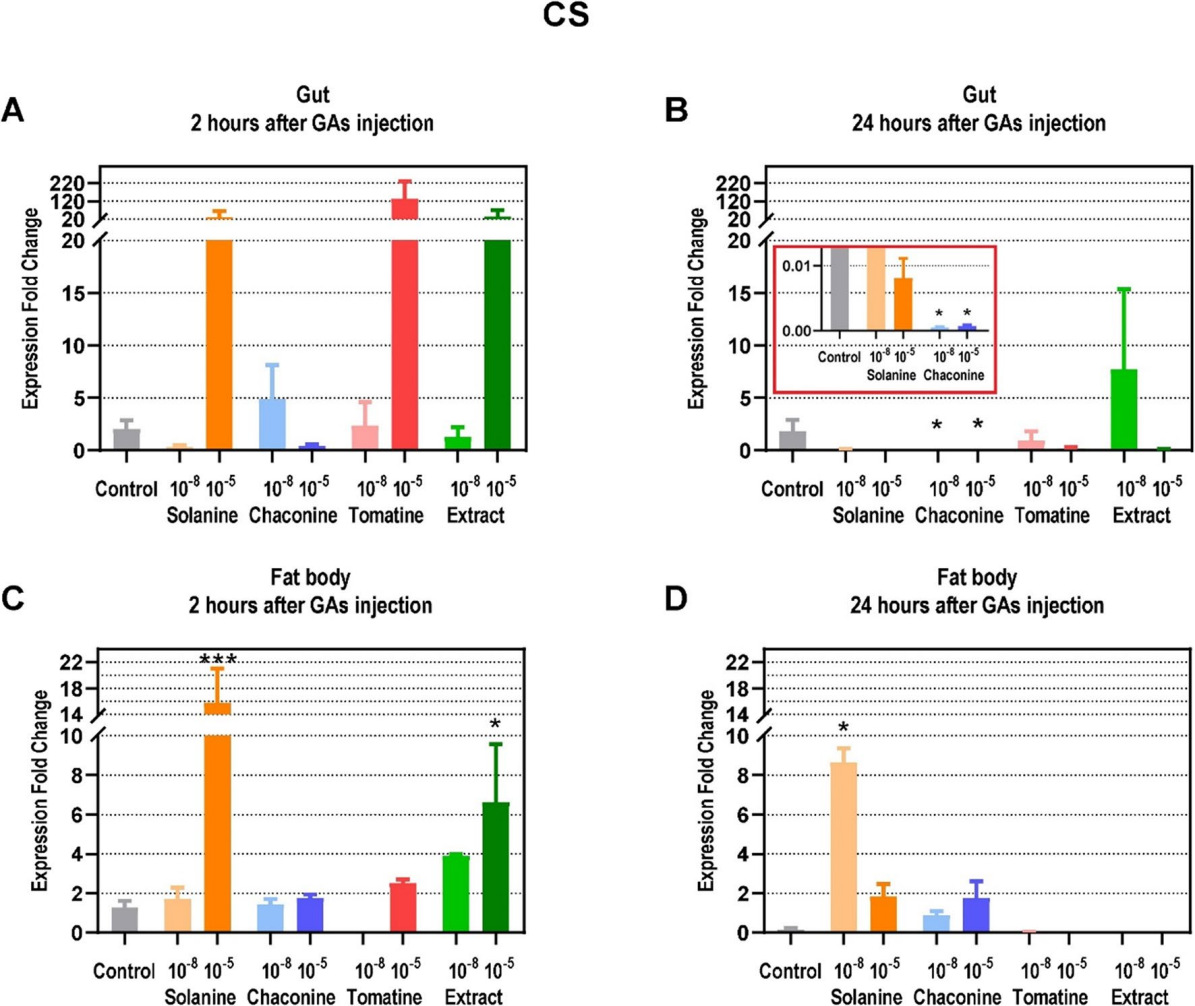
Expression fold change of citrate synthase (CS) in a gut (**A**, **B**) and fat body (**C**, **D**) of *Tenebrio molitor* larvae 2 and 24 h after application of 10^–8^ and 10^–5^ M solutions of glycoalkaloids and physiological saline (control) compared to the L ribosomal proteins (RPL) expression. Data are shown as mean ± SEM. The analysis was performed in triplicate. The pooled samples were used with *n* = 5. Data are compared with Kruskal–Wallis with Dunn's multiple comparison test. Statistically significant differences between tested groups and the control are indicated with asterisks, *** *p* ≤ 0.001, * *p* ≤ 0.05

There was a tendency for the expression of the CS encoding gene to increase in the gut 2 h after higher concentrations of SOL, TOM, and EXT injection, but the changes were not significant (Fig. 5A). On the contrary, CHA treatment resulted in a considerable decrease in *CS* expression after 24 h in this tissue (Fig. 5B). In the fat body, there was also an increase in *CS* expression 2 h after 10^–5^ M SOL and EXT treatment reported (Fig. 5C). SOL at lower concentration increased the CS gene expression 24 h after injection in this tissue 53-fold (Fig. 5D). Other GAs did not affect the gene expression compared to the control.

**Fig. 6:**
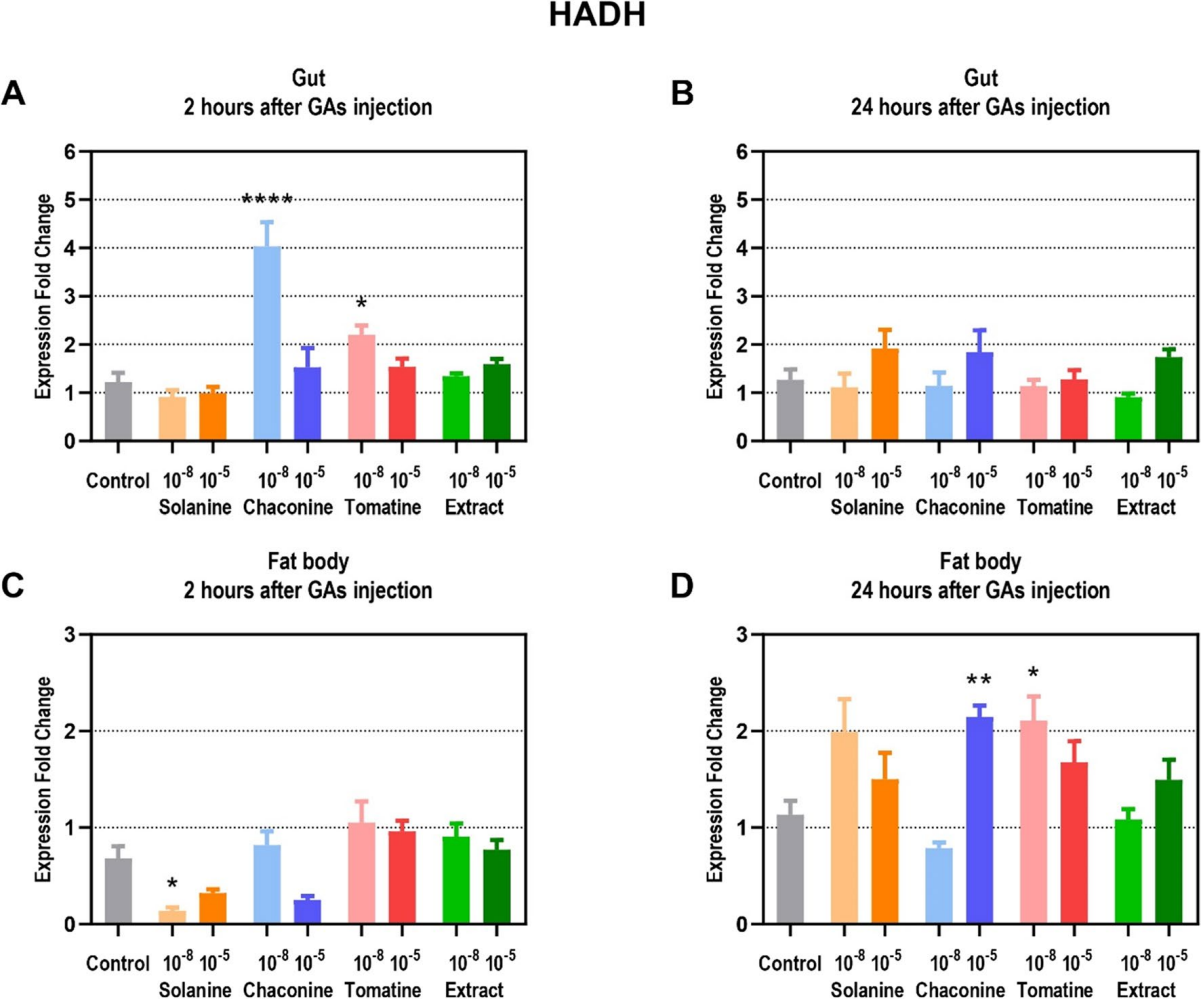
Expression fold change of β-hydroxyacyl-CoA dehydrogenase (HADH) in a gut (**A**, **B**) and fat body (**C**, **D**) of *Tenebrio molitor* larvae 2 and 24 h after application of 10^–8^ and 10^–5^ M solutions of glycoalkaloids and physiological saline (control) compared to the L ribosomal proteins (RPL) expression. Data are shown as mean ± SEM. The analysis was performed in triplicate. The pooled samples were used with *n* = 5. Data are compared with Kruskal–Wallis with Dunn's multiple comparison test. Statistically significant differences between tested groups and the control are indicated with asterisks, *** *p* ≤ 0.001, ** *p* ≤ 0.01, * *p* ≤ 0.05

The expression of the genes encoding HADH increased in the gut 2 h after a lower concentration of CHA and TOM injections more than 3 times and almost 2 times, respectively (Fig. 6A). After 24 h, no significant changes in protein gene expression were observed in this tissue with the expression fold change ranging between 0.9 ± 0.22 and 1.9 ± 1.18 (Fig. 6B). In the fat body, *HADH* expression decreased almost 5 times 2 h only after SOL 10^–8^ M treatment (Fig. 6C). On the contrary, an almost twofold increase in HADH gene expression was reported 24 h after 10^–5^ M CHA as well as after 10^–8^ M TOM application (Fig. 6D).

### Enzyme activity

**Fig. 7:**
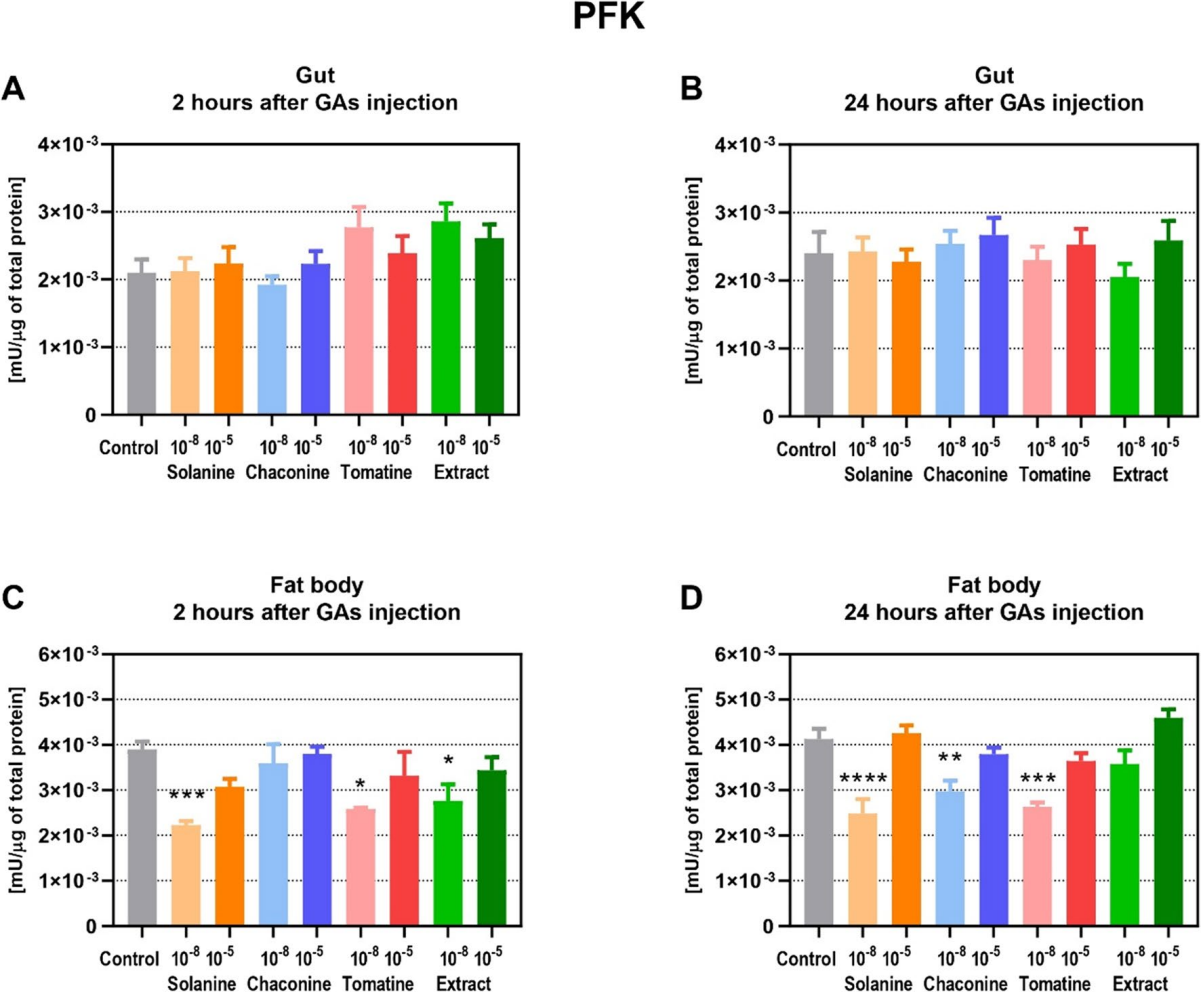
The activity of phosphofructokinase-1 (PFK) in the gut (**A**, **B**) and fat body (**C**, **D**) of *Tenebrio molitor* larvae 2 and 24 h after injection with glycoalkaloids and physiological saline (control). The concentrations of the compounds 10^−8^ M (10^−8^) and 10^−5^ M (10^−5^) are presented above. The enzyme activity is expressed as mU per μg of total soluble protein in the sample. Data are shown as mean ± SEM. The assay was performed in triplicate. The pooled samples were used with *n* ≥ 10. Data are compared with ordinary one-way ANOVA with Dunnett's multiple comparison test. Statistically significant differences between tested groups and the control are indicated with asterisks, **** *p* ≤ 0.0001, *** *p* ≤ 0.001, ** *p* ≤ 0.01, * *p* ≤ 0.05

The activity of PFK and CS was also analyzed in the gut and fat body after GAs injections. The PFK activity was not affected by the tested compounds neither 2 nor 24 h after the GAs application (Fig. 7A, B). The PFK activity 2 h after GAs injection ranged between 1.9 × 10^–3^ ± 0.69 × 10^–3^ and 2.9 × 10^–3^ ± 1.47 × 10^–3^ mU/μg, while 24 after GAs treatment, values between 2.1 × 10^–3^ ± 1.05 × 10^–3^ and 2.7 × 10^–3^ ± 1.40 × 10^–3^ mU/μg of total soluble protein in the samples were determined. On the contrary, PFK activity decreased in the fat body 2 h after injection with the lower concentration of SOL, TOM, and EXT (Fig. 7C). The lowest enzyme activity was observed after 10^–8^ M SOL treatment (almost a twofold decrease compared to the control). A decrease in PFK activity was also reported 24 h after the application of most GAs tested in lower concentrations: SOL, CHA, and TOM (Fig. 7D). Therefore, GAs injection caused a decrease in PFK activity in the fat body, while it did not change protein activity in the insect gut.

**Fig. 8:**
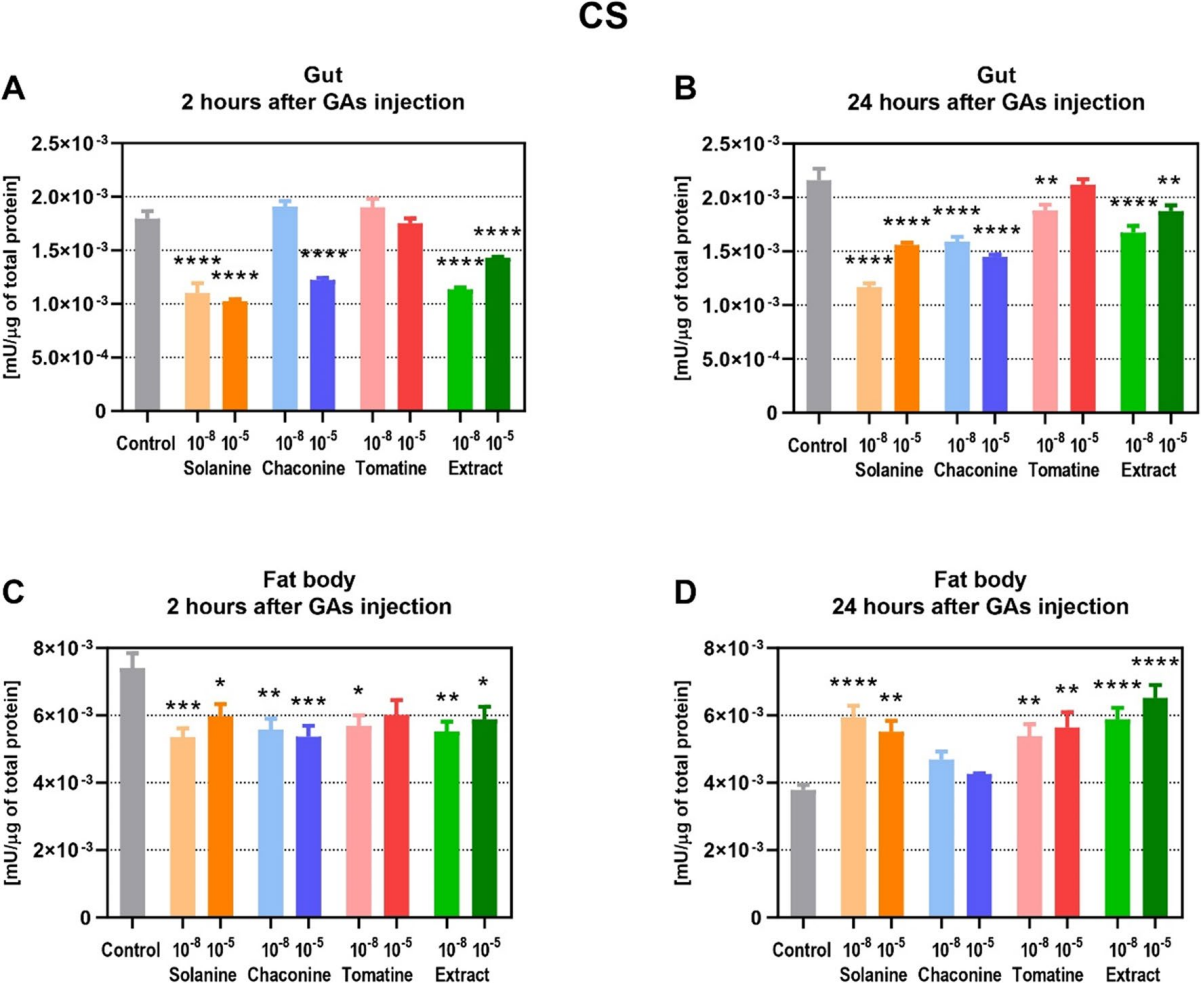
The activity of citrate synthase (CS) in the gut (**A**, **B**) and fat body (**C**, **D**) of *Tenebrio molitor* larvae 2 and 24 h after injection with glycoalkaloids and physiological saline (control). The concentrations of the compounds 10^−8^ M (10^−8^) and 10^−5^ M (10^−5^) are presented above. The enzyme activity is expressed as mU per μg of total soluble protein in the sample. Data are shown as mean ± SEM. The assay was performed in triplicate. The pooled samples were used with *n* ≥ 10. Data are compared with ordinary one-way ANOVA with Dunnett's multiple comparison test. Statistically significant differences between tested groups and the control are indicated with asterisks, **** *p* ≤ 0.0001, *** *p* ≤ 0.001, ** *p* ≤ 0.01, * *p* ≤ 0.05

Treatment with SOL, EXT and 10^–5^ M CHA resulted in decreased CS activity in the gut after 2 h (Fig. 8A). The observed changes were also maintained 24 h after injection in that tissue (Fig. 8B). Additionally, CHA 10^–8^ M and TOM 10^–8^ M also caused similar changes. Almost in all experimental variants in the fat body, the CS activity was lower compared to the control (Fig. 8C). Surprisingly, the SOL, TOM and EXT treatment resulted in increased enzyme activity 24 h after injection (Fig. 8D). Therefore, the results indicate that the CS activity after GAs injections decreased in the gut at both the incubation time tested and in the fat body 2 h after treatment. Interestingly, the CS activity was increased 24 h after GAs application in the fat body, except for CHA.

## Discussion

In the study, the effects of SOL, CHA, TOM and EXT on the energy metabolism of the larvae of *T. molitor* beetle were studied. For this purpose, the level of energy metabolites in insect tissues, as well as the level of gene expression and the activity of the key enzymes of glycolysis, the TCA cycle and fatty acids β-oxidation, were determined 2 and 24 h after GAs injection.

The main reserve of the energy substrates in insects are glycogen and TAGs. The effect of GAs on the TAGs content in *T. molitor* larvae has previously been reported (Winkiel et al. 2023). The glycogen is composed of glucose moieties, and it is stored in the fat body adipocytes. The tested GAs did not alter neither glycogen nor glucose concentration in that tissue (Fig. 1E-H). In the haemolymph, these carbohydrates were not detected in our study. In contrast, the increase in glycogen concentration in the fat body of *T. molitor* after solamargine application and the reduction of its content after *S. nigrum* extract treatment were evidenced (Spochacz et al. 2018). The trehalose content did not change in the fat body, as a result of GAs injection (Fig. 1C, D). However, the concentration of this main circulating carbohydrate was reduced in the haemolymph 2 h after TOM treatment (in the form of pure GA and as the extract), while it increased after 24 h in these experimental variants (Fig. 1A, B). The decrease in trehalose content in haemolymph may be the result of the energy requirement for GAs detoxification, which leads to increased nutrient catabolism. Additionally, GAs increase oxidative stress in insects (Adamski et al. 2014; Winkiel et al. 2024). As trehalose has the ability to scavenge the hydroxyl radical and decrease the reactive oxygen species (ROS) content (Felton and Summers 1995), the later rise of this disaccharide concentration may help alleviate the effect of increased ROS production. Moreover, trehalose may be synthesized in insects from free fatty acids (McDougall and Steele 1988), and, in fact, it was previously reported that GAs, especially after 24 h, increase the level of these compounds in the *T. molitor* beetle (Winkiel et al. 2023). On the other hand, in previous studies on *G. mellonella* larvae (Spochacz et al. 2021) it was observed that solasonine and *S. nigrum* extract did not affect trehalose concentration in the insect haemolymph, which may indicate on the different mechanisms of action of the tested GAs depending on the insect species, or the structure and concentration of the GA compound. The application of GAs is especially important because the insects in other mentioned studies were not injected but fed with the addition of GAs, which certainly translates into distribution and metabolism of GAs (Spochacz et al. 2018, 2021).

**Fig. 9:**
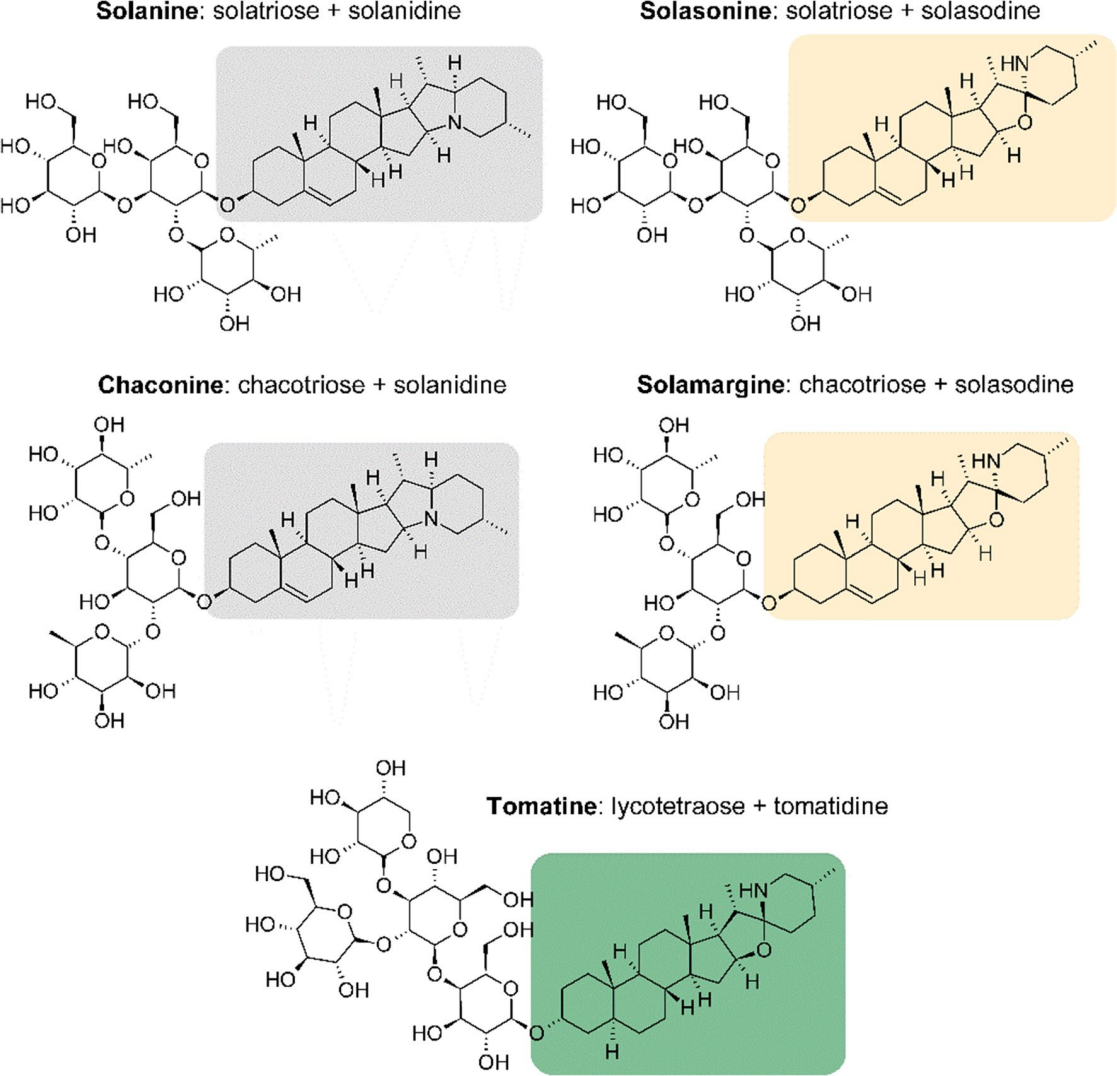
Structures of *Solanaceae* glycoalkaloids (Winkiel et al. 2022)

The synthesis of amino acids takes place mainly in the fat body. They may be derived from glucose or acetate, which can be incorporated to the intermediates of the glycolysis or TCA cycle. Then, the amino group is added in the transamination reaction or by adding ammonia (Chapman 2012). Besides the role of amino acids in synthesis of proteins, which are involved in transport, signalling, gene expression, membrane activities, as well as acting as enzymes, these compounds have additional functions related to neurotransmitters synthesis, detoxification, and ATP production (Castagna et al. 1997; Manière et al. 2020). However, some amino acids, which are considered as essential for larval growth (Chen 1966; Davis 1975), have not been detected in this research (arginine, histidine, lysine, tryptophan, methionine, threonine, isoleucine). This intriguing finding may be related to their trace amounts in analyzed tissues. It might also be explained by the fact that these amino acids were present at a higher level in the tissues that were not analyzed in this research. The following amino acids were detected in the tested tissues: valine, leucine, proline, phenylalanine, and tyrosine in the fat body (Fig. 3) while valine, leucine, proline, phenylalanine, and alanine in the haemolymph (Fig. 2). The obtained results indicate to the possible transport of most of the identified amino acids from haemolymph to the fat body, especially 24 h after pure TOM and EXT injection (Fig. 3). Their accumulation in that tissue may be the result of the protein degradation after TOM treatment. However, it seems more reasonable that increased synthesis of non-essential amino acids (proline, tyrosine) serves as an energy source for the severe detoxification processes after 24 h since GAs application. On the other hand, tyrosine is the amino acid necessary for the sclerotization of the cuticle (Andersen 2010), usually synthesized from phenylalanine (Vavricka et al. 2014). Therefore, the increased content of both amino acids after GAs injection may impact the sclerotization process. The most important amino acid used for ATP production, for example, during flight, is proline. It is also the key component of antimicrobial peptides (Yi et al. 2014) and plays an important role in the tolerance of cold in insects (Misener et al. 2001; Lubawy et al. 2022). In the haemolymph, the decrease in proline concentration was reported, and these results are consistent with the study that describes the decrease in proline level observed after *S. nigrum* extract and solasonine in larvae of *G. mellonella* (Spochacz et al. 2021). Alanine is the amino acid used for proline synthesis (Arrese and Soulages 2010). Therefore, alanine concentration was also decreased in the haemolymph 24 h after EXT injection. The other detected amino acids may constitute an additional energy source with a high potential amount of ATP, which could be generated during the oxidation (Bursell 1981). Furthermore, the obtained results showed that the mechanisms of SOL and CHA action are different from TOM, because, after their injection, no amino acid accumulation was observed in the fat body tissue. The explanation may be the structures of GAs (Fig. 9). SOL and CHA are composed of solanidine and three carbohydrate molecules, while TOM contains tomatidine skeleton and four carbohydrate moieties (Nepal and Stine 2019, Winkiel et al. 2022), which may affect their properties and mechanisms of action in insect tissues.

The energy substrates are utilized in the processes that produce ATP. Changes in the glycolysis process have an impact on cell survival and growth, although it does not produce much energy compared to oxidative phosphorylation (Xu et al. 2022). Therefore, this pathway might be the target of anticancer therapy. For instance, SOL exhibited anticancer effects via the regulation of glycolysis pathway in non-small cell lung cancer (Zou et al. 2022) as well as in human renal cancer (Wang et al. 2021). It was reported that some plant secondary metabolites inhibit glycolysis enzymes activity which may lead to the cell apoptosis. For example, sesquiterpenes might decrease PFK activity, the key regulatory enzyme of glycolysis, in animal cells (Morrissey 2009). Furthermore, coumarin, a secondary plant metabolite, inhibited glycolysis in the *Spodoptera litura* F. moth (Xia et al. 2023). On the other hand, the GA extract of *S. tuberosum* activated the glycolytic pathway in *Fusarium solani* (Mart.) Sacc (Zhang et al. 2023). It reduced the amount of glucose in the fungus cells and the activity of hexokinase, while increasing PFK and pyruvate kinase activity. The presented study showed that GAs did not affect PFK activity in the insect gut (Fig. 7A, B). However, they decreased enzyme activity in the fat body, which may indicate inhibition of glycolysis (Fig. 7C, D). The changes after GAs injections were also found at the gene expression level, but only 24 h after treatment. TOM and EXT reduced the expression of PFK-encoding genes in the gut (Fig. 4B). On the other hand, SOL and EXT increased this parameter in the insect fat body (Fig. 4D), which may be the compensatory effect of decreased enzyme activity. Increased *PFK* expression in the fat body may also indicate increased intensity of glycolysis in order to intensify ATP production. Similar effects of increased *PFK* expression level were also observed in *Hyphantria cunea* D. moth after coumarin treatment (Yuan et al. 2024). Therefore, the *PFK* expression as well as the enzyme activity were affected by GAs treatment, but the analyzed parameters may differ depending on the compound and the tested tissue. The pyruvate, a product of glycolysis, usually enters the mitochondria where it is oxidized to acetyl-CoA in the TCA cycle. Unfortunately, there is no literature that describes the effects of GAs on the expression level of the genes that encode CS in insects, as well as the activity of this protein, which is the crucial enzyme of the TCA cycle. However, coumarin was recently reported to inhibit TCA cycle pathways in *S. litura* moth at the gene expression level (Xia et al. 2023). Furthermore, this plant secondary metabolite affects energy metabolism in *H. cunea* moth, decreasing the larval nutrient content and the expression of genes involved in the mentioned process (Yuan et al. 2024). This finding is consistent with the results obtained in this work, because GAs already after 2 h decrease CS activity in the insect gut (Fig. 8A, B), but significant changes at the gene level were visible only 24 h after CHA treatment (Fig. 5B). Also in the fat body, 2 h after GAs application a decrease in CS activity was reported (Fig. 8C). However, later, this parameter increased compared to the control in that tissue (Fig. 8D), and these results correspond to the increased expression of genes encoding CS after SOL and EXT application (Fig. 5C, D). It may indicate an increase in the number of mitochondria and an increase in the oxidative capacity of cells. The explanation could also be the increased energy demand for stress response and GAs detoxification pathways (du Rand et al. 2015) .

Next to pyruvate, other important energy substrates are fatty acids, which may be converted during β-oxidation to acetyl-CoA, the substrate of the Krebs cycle. Camptothecin in the fat body of *Spodoptera frugiperda* S. was recently observed to affect the expression of important genes involved in the synthesis of fatty acids. Furthermore, changes in the expression of genes related to the lipid biosynthesis pathway and lipid metabolites after SOL treatment were also found in the *Curvularia trifolii* K. fungus (Xu et al. 2023). One of the enzymes involved in fatty acid oxidation is HADH. It was found to play a crucial role in lipid mobilization in insects (Arêdes et al. 2022). Our results showed that the level of expression of the genes encoding HADH is higher 2 h after CHA and TOM treatment in the gut (Fig. 6A). However, in the fat body this parameter is reduced (Fig. 6C), and this accords with our previous observations, which showed decreased HADH activity 2 h after SOL and TOM application (Winkiel et al. 2023). Interestingly, after 24 h, HADH activity in the fat body is reduced, while the expression level increases (Fig. 6D). Therefore, the results indicated a possible reversed correlation between protein expression and enzyme activity. Increased gene expression could be a compensatory mechanism for reduced enzyme activity. Thus, GAs, as other plant secondary metabolites, may alter the lipid metabolism also at the gene level.

## Conclusions

The present study was designed to determine the effect of GAs on the concentration of energy substrates and on the energy metabolism processes in the tissues of *T. molitor*. The research has shown that TOM and EXT affect the trehalose concentration in the insect haemolymph. They also lead to the accumulation of most of the amino acids detected after 24 h in fat body tissue, reducing their content in the haemolymph, thus, suggesting possible transport of amino acids between tissues. This effect was not observed after SOL and CHA treatment, which indicates the different mechanisms of action. The observed changes may be the result of protein degradation and/or enhanced catabolism reactions for ATP production as an energy source for detoxification processes. The tested GAs also affect glycolysis, TCA cycle, as well as fatty acids β-oxidation pathways regulating the activity and gene expression of key enzymes of these processes, but the effect depends on the type of GA compound, the type of the tested tissue, and the incubation time after treatment. Furthermore, the study revealed possible compensatory mechanisms related to the reduced activity of the enzymes tested after application of GAs, which resulted in an increased level of expression of *PFK* and *HADH*. On the other hand, the inhibited TCA pathway was reported in the insect gut, while an enhanced process was noted in the fat body. Taken together, these results suggest that GAs affect the energy metabolism of *T. molitor*. The study contributes to our understanding of the mechanisms of the activity of plant secondary metabolites in insects. As ATP production is necessary for the survival of the organism, this knowledge may facilitate the design of new natural biopesticides against insect pests. However, further research should be undertaken in this topic. For example, investigating the effects in other insect species as well as using longer incubation times would allow for an even more comprehensive scheme of action of the tested plant metabolites in animals.

## Supplementary Information

Below is the link to the electronic supplementary material.

Supplementary file1 (DOCX 12 KB)

## Data Availability

The data analysed during this study are included in this published article.

## Abbreviations

CHA: α-Chaconine
CS: Citrate synthase
EXT: Tomato leaf extract
Gas: Glycoalkaloids
GC-MS: Gas chromatography ‒ mass spectrometry
HADH: β-Hydroxyacyl-CoA dehydrogenase
PFK: Phosphofructokinase-1
ROS: Reactive oxygen species
SOL: α-Solanine
TAGs: Triglycerides
TCA: Tricarboxylic acid
TOM: α-Tomatine
