# Supplementary material for: A tomato a day keeps the beetle away – the impact of Solanaceae glycoalkaloids on energy management in the mealworm Tenebrio molitor
Source: Environ Sci Pollut Res Int. 2024 Sep 25;31(48):58581–98. doi: 10.1007/s11356-024-35099-4 (PMC11467077; doi:10.1007/s11356-024-35099-4)
Supplement: Supplementary file 1 — Supplementary file1 (DOCX 12 KB) [file 11356_2024_35099_MOESM1_ESM.docx]

Table 1. Primers sequences used in qPCR

| Name | Sequence | Amplicon length |
| --- | --- | --- |
| RPL13a-F | TCGTCGTGAGATGCGAACAA | 191 bp |
| RPL13a-R | CTGCTTCCCACGTTCTGTCT |  |
| PFK-F | TCTCATTCAAAGCGGTGTCA | 167 bp |
| PFK-R | GTTAATCATTGGCGGTTTCG |  |
| CS-F | ATATCGAAACTTCCCGTTGC | 176 bp |
| CS-R | TGGTCAGCGTGGATCACTAA |  |
| HADH-F | CTCCCGGATTCATTGTCAAC | 161 bp |
| HADH-R | GACCGACGTAATCGGACAAT |  |
